# Analysis of potential impact factors of corneal biomechanics in myopia

**DOI:** 10.1186/s12886-023-02891-8

**Published:** 2023-04-06

**Authors:** Yangrui Du, Yuqing Zhang, Yu Zhang, Tao Li, Jie Wang, Zhiyu Du

**Affiliations:** 1 Department of Ophthalmology, The Second Affiliated Hospital of Chongqing Medical University, Chongqing, 400010 People’s Republic of China; 2 Medal Eye Institute, Chongqing, China

**Keywords:** Myopia, Corneal biomechanical, Central corneal thickness, Spherical equivalence

## Abstract

**Purpose:**

To investigate potential impact factors associated with corneal biomechanical properties in Chinese myopia and further to investigate quantifying corneal biomechanics in clinical work.

**Methods:**

Three hundred fifty-five eyes from 181 healthy myopic subjects with a mean age of 25.1 ± 9.4 were recruited in this study. Each patient carried out a comprehensive ophthalmic examination, including corneal hysteresis(CH), corneal resistance factor(CRF), central corneal thickness(CCT), axial length(AL), intraocular pressure(IOP), spherical equivalence(SE) and corneal curvature (K). CH and CRF were measured with the ocular response analyzer(ORA). To analyze the corneal biomechanical characteristics of myopia patients and their association with other parameters.

**Result:**

The multiple linear regression analysis showed that CH was positively associated with CCT, and corneal curvature (all with *P* < 0.05) and negatively associated with SE and AL)(all with *P* < 0.05); CRF was positively correlated with CCT, corneal curvature and IOP(all with *P* < 0.05), but negatively correlated with SE and AL(all with *P* < 0.05). The CH and CRF values in children group were both higher than adults group (≥ 18 years old) (*P* < 0.05), but it attributed to that the CCT of children was thicker than adults. Excluding factor of CCT, there was no significant difference in CH and CRF between children group and adult group. Excluding factor of CCT, there was no significant difference in CH and CRF among different stage of age (age 18–48).

**Conclusion:**

The CCT played the most important role of affecting the CH and CRF. The SE, corneal curvature, AL and IOP had a certain influence on corneal biomechanics. Whether the CH and CRF values of individual patient are normal in clinical work, it should refer to the CH and CRF values corresponding CCT sectional range and SE.

## Introduction

The cornea is a mechanical barrier layer mainly composed of viscoelastic tissue, with certain hardness and elasticity, and its integrity maintains relatively constant intraocular pressure. To objectively evaluate the hardness and elasticity of normal and abnormal corneas, not only contributes to investigate the effect of ophthalmopathy on corneal biomechanics, but also provides a reference basis for research on changes of biomechanical properties after corneal refractive surgery.

Corneal refractive surgery has shown safety, stability and predictability when operated on patients after decades of development, particularly with the development of more advanced laser refractive surgery. However, how to exclude potential keratoconus and other effects of ophthalmopathy in the preoperative screening is also a challenge for ophthalmologists, preoperative thin corneas and thinner residual stromal bed after excimer laser ablation are widely described as risk factors for the postoperative corneal ectasia. Keratoconus is an ectatic, noninflammatory disorder in which corneal thinning and protrusion cause the cornea to become a conical shape. Highly irregular astigmatism is the main performance in patients with keratoconus, and it will seriously influence vision. The descending of corneal biomechanics has a close relationship with the development of keratoconus. As we know, the Ocular Response Analyzer (ORA; Reichert Ophthalmic Instruments, Buffalo, New York, USA) has allowed ophthalmologists to measure the biomechanical properties of the cornea quantitatively, such as corneal hysteresis(CH) and corneal resistance factor(CRF). The ORA determines corneal biomechanical metrics using the cornea was subjected to the process of compression, first applanation, concaving cornea, second applanation and returning to its original state, and the changes of corneal shape and airflow pulse pressure with time were accurately recorded and analyzed to reflect the characteristics of corneal biomechanical properties. CH is described to predominantly reflect the viscous properties of the cornea and CRF is a synthetic measure representative of corneal elastic properties [1].

Up to now, many excellent international teams have focused on researching the biomechanical properties of cornea measured by the ORA and their associations with central corneal thickness(CCT), spherical equivalence(SE), corneal curvature(K), axial length(AL), intraocular pressure(IOP) and age [2–5]. Corneal biomechanics has become a hot topic related with keratoconus, corneal ectasia and other ophthalmopathy. However, there are still many controversies existing regarding the relationship between corneal biomechanical properties and age [6–9]. While some studies showed the CH and CRF decreased with advancing age [9], a few articles reported that there was no statistical difference between corneal biomechanical properties and age [6, 7]. These disputes will arouse us to think several problems. Whether the CH and CRF values of individual patient are normal in clinical work, we only refer to the mean CH and CRF values, or we also should refer to the other index? What factors will affect the CH and CRF values? How much are the variational ranges of the CH and CRF values under the influence of the factors? In order to solve these problems, we aimed to investigate the potential impact factors that affected corneal biomechanical properties in this retrospective study.

## Materials and methods

### Study patients

This is a retrospective study designed to investigate associations between corneal biomechanical properties and potential impact factors in Chinese myopia, selecting clinical information of total 355 eyes (178 left eyes, 177 right eyes) from 181 myopia aged 8 to 57 years(mean age of 25.1 ± 9.4) who visited Medal Eye Institute between february 2014 and february 2016.

### Data collection

Each patient carried out a comprehensive ophthalmic examination, including assessment of best corrected visual acuity (BCVA), assessment of corneal biomechanical properties by the ORA measurements (Reichert Ophthalmic Instruments, Buffalo, New York, USA), measurement of the central corneal thickness (CCT) and axial length (AL) by the ultrasonic pachymeter (Sonomed Inc. 1979 Marcus Avenue Lake Success, NY 11,042, USA). The measurement of the intraocular pressure (IOP), spherical equivalence (SE) and corneal curvature by the Schiotz tonometer, the Retinoscopy instrument and Corneal Topography(CSO, Firenze, Italy) respectively. IOP and CCT were measured for each patient at the same time, between 3 and 5 PM each day, and IOP was measured each time before CCT was measured. Three measurements were taken in each eye, and the mean of the measurements was used in the analysis. None of the included eyes had any ocular disease except a refractive error. Myopia was defined as at least 0.5 diopters (D) of spherical equivalent refraction (SER). Subjects with contact lens used, pathological changes of cornea as opacities scars, eyes with history of ocular surgery(included refractive surgery), history of ocular trauma, glaucomatous eyes were excluded. Patients with systemic disease (such as diabetes or hypertension) were also excluded. All these examinations were conducted by a single ophthalmologist.

Patients were grouped according to age as follows: group I(8 ≤ group I < 18 years old), group II(18 ≤ group II < 28 years old), group III(28 ≤ group III < 38 years old) and group IV(38 ≤ group IV ≤ 48 years old). Meanwhile, we set the 355 eyes as low, moderate, and high myopic groups according the diopter.

### Statistical analysis

Statistical management and analysis were conducted using SPSS version 19.0 statistical package (SPSS, Chicago, Illinois, USA). Independent sample t-tests were used to assess if each parameter had a normal distribution. Multiple linear regression models were composed with CH and CRF as the dependent variables and CCT, SE, corneal curvature, AL, IOP as the covariates. Kruskal–Wallis test and paired t -test were used for intergroup comparisons.The results were considered statistically significant at a P value less than 0.05 for the CH and CRF.

## Results

Three hundred and fifty-five eyes of 181 subjects were included in the study after excluding some eyes according to the exclusion criteria.

The associations between corneal biomechanical properties and potential impact factors were examined through linear regression analyses and multivariate models (Table 1). This study showed that CH was negatively associated with SE and AL (regression coefficient =  − 0.185 and − 0.142, resp.; all with *P* < 0.05) and positively associated with CCT and corneal curvature (regression coefficient = 0.649 and 0.174, resp.; all with *P* < 0.05). CRF was negatively associated with SE and AL (regression coefficient =  − 0.109 and − 0.166, resp.; all with *P* < 0.05). CRF was positively associated with CCT (regression coefficient = 0.590, *P* < 0.05), and IOP (regression coefficient = 0.236, *P* < 0.05) and corneal curvature (regression coefficient = 0.156, *P* < 0.05) (Table 1). In summary, the multiple linear regression analysis showed that CH and CRF increased accompanied by CCT and corneal curvature increasing, CH and CRF reduced accompanied by the spherical equivalence and axial length increasing.

**Table 1:** Multiple linear regression analysis on corneal hysteresis and corneal resistance factor

	CH			CRF		
	Partial regression coefficient	Standardized partial regression coefficient	*P* value	Partial regression coefficient	Standardized partial regression coefficient	*P* value
SE	-0.078	-0.185	< 0.05	-0.109	-0.109	< 0.05
Corneal curvature	0.171	0.174	< 0.05	0.184	0.156	< 0.05
CCT	0.024	0.649	< 0.05	0.026	0.590	< 0.05
IOP	-	-	< 0.05	0.236	0.236	< 0.05
AL	-0.138	-0.142	< 0.05	-0.191	-0.166	< 0.05

*Abbreviations*: *CH* Corneal hysteresis, *CRF* Corneal resistance factor, *SE* Spherical equivalence, *CCT* Central corneal thickness, *IOP* Intraocular pressure, *AL* Axial length

Multiple linear regression analysis has showed the association between CH, CRF and CCT, SE, AL, IOP, corneal curvature. However, the associations of age with corneal biomechanical properties have been argued all the way. In order to probe into the argument, our study summarized the mean CH in children group(mean age ± standard deviation 13.85 ± 2.81) and adults group(mean age ± standard deviation 27.56 ± 8.50) were 11.12 ± 1.20 and 10.26 ± 1.34 mm Hg respectively, while the CRF in children group and adults group were 11.00 ± 1.69 and 10.20 ± 1.64 mm Hg respectively. Obviously, the values of CH and CRF in children group were both higher than adults group (Table 2).

**Table 2:** Differences of the mean CH and CRF in children group and adults group

	Children group (65 eyes)	Adults group (290 eyes)	Z	*P* value
Age (year)	13.85 ± 2.81	27.56 ± 8.50		
CH(mmHg)	11.12 ± 1.20	10.26 ± 1.34	-4.513	0.000
CRF(mmHg)	11.00 ± 1.69	10.20 ± 1.64	-3.442	0.001

Kruskal–Wallis test, statistic value: Z. *P* < 0.05 represented statistical significance

*Abbreviations*: *CH* Corneal hysteresis, *CRF* Corneal resistance factor

However, children group(range, 561.26 ± 38.61um) was found to have significantly thicker CCT than did the adults group (range, 538.92 ± 35.98um). So we selected 62 eyes which CCT has no significant difference each from children group and adults group respectively. We set that as new children group and new adults group (Table 3), and we obtained an mean CH of 11.06 ± 1.19 mmHg in the new children group and 10.90 ± 1.12 mmHg in the new adults group. While the CRF in new children group and new adults group were 10.87 ± 1.60 and 10.95 ± 1.34 mmHg respectively. Meanwhile, we found that there were no significant differences about CH and CRF in new children group and new adults group after adjusting for CCT. Obviously, these results suggested that age was not an independent influence factor of CH and CRF. In order to further explore the role of age in corneal biomechanics, we grouped patients according to age as follows: group I(8 ≤ group I < 18 years old), group II(18 ≤ group II < 28 years old), group III(28 ≤ group III < 38 years old) and group IV(38 ≤ group IV ≤ 48 years old).The data of CH, CRF and CCT were statistically analyzed in groups of different age (Mean ± SD) (Table 4). And we found that there were no significant differences about CH and CRF of different stage of age(from 18 to 48 years old). Meanwhile, CCT there was no statistical difference between different stage of age(from 18 to 48 years old) (Table 5). As stated above, the result of research shows that the CCT and SE had great effect on corneal biomechanical properties. However, how to combine the CCT and SE to judge the quality of corneal biomechanics is still lack of corresponding reference standard for clinicians. So our study summarized that the mean CH in CCT < 500 μm, 500-540 μm, 540-580 μm, > 580 μm groups were 8.76 ± 1.08, 9.94 ± 1.11, 10.82 ± 1.10 and 11.77 ± 1.04 mmHg respectively, while CRF were 8.48 ± 1.16, 9.55 ± 1.11, 10.86 ± 1.24 and 12.26 ± 1.40 mm Hg respectively (Table 6). It is obvious that as the thickness of cornea increased, the CH and CRF increased. Meanwhile, we set the 355 eyes as low, moderate, and high myopic groups according the diopter, and found that the CH and CRF in the high myopic group were significantly lower than in the low myopic (*P* < 0.05) or moderate (*P* < 0.05) group (Table 7). Obviously, this result indicated that as the diopter increased, CH and CRF decreased.

**Table 3:** Differences of the mean CH and CRF in new children group and new adults group

	New children group (62 eyes)*	New adults group (62eyes)*	t	*P* value
CH(mmHg)	11.06 ± 1.19	10.90 ± 1.12	0.867	0.389
CRF(mmHg)	10.87 ± 1.60	10.95 ± 1.34	-0.427	0.671

Paired t -test. *Excluding factor of CCT. *P* < 0.05 represented statistical significance

*Abbreviations*: *CCT* Central corneal thickness, *CH* Corneal hysteresis, *CRF* Corneal resistance factor

**Table 4:** The comparative analysis of CH, CRF, CCT in groups of different age (Mean ± SD)

	Group I	Group II	Group III	Group IV
CH (mmHg)	11.12 ± 1.20	10.22 ± 1.44	10.25 ± 1.32	10.41 ± 1.28
CRF (mmHg)	11.01 ± 1.69	10.25 ± 1.68	9.95 ± 1.40	10.37 ± 1.97
CCT(μm)	561.26 ± 38.61	538.78 ± 37.37	537.47 ± 32.36	534.10 ± 36.08

8 ≤ Group I < 18 years old; 18 ≤ Group II < 28 years old; 28 ≤ Group III < 38 years old; 38 ≤ Group IV ≤ 48 years old

*Abbreviations*: *CCT* Central corneal thickness, *CH* Corneal hysteresis, *CRF* Corneal resistance factor

**Table 5:** The comparative analysis of CH, CRF, CCT among different age groups each other

Variables	*P* value					
	I vs II	I vs III	I vs IV	II vs III	II vs IV	III vs IV
CH(mmHg)	0.000	0.000	0.017	0.851	0.470	0.590
CRF(mmHg)	0.002	0.000	0.081	0.187	0.699	0.228
CCT(μm)	0.000	0.000	0.001	0.794	0.510	0.666

8 ≤ Group I < 18 years old; 18 ≤ Group II < 28 years old; 28 ≤ Group III < 38 years old; 38 ≤ Group IV ≤ 48 years old

*Abbreviations*: *CCT* Central corneal thickness, *CH* Corneal hysteresis, *CRF* Corneal resistance factor

**Table 6:** Differences of CH and CRF in different CCT groups

	CCT(μm)				F	*P*-value
	< 500	500–540	540–580	> 580		
CH(mmHg)	8.76 ± 1.08*	9.94 ± 1.11*	10.82 ± 1.10*	11.77 ± 1.04*	77.519	0.000
CRF(mmHg)	8.48 ± 1.16*	9.55 ± 1.11*	10.86 ± 1.24*	12.26 ± 1.40*	105.967	0.000

*Abbreviations*: *CH* Corneal hysteresis, *CRF* Corneal resistance factor

^*^Comparision among the four groups each other LSD-t *P* < 0.05

**Table 7:** Differences of the mean CH and CRF in the low, moderate and high myopic groups

	SE(D)			F	*P*-value
	Low myopia	Moderate myopia	High myopia		
CH (mmHg)	10.20 ± 0.99*	10.31 ± 1.09*	9.67 ± 1.14*	5.412	0.006
CRF(mmHg)	10.03 ± 0.91*	9.96 ± 1.19*	9.18 ± 0.96*	8.649	0.000

*Abbreviations*: *CH* Corneal hysteresis, *CRF* Corneal resistance factor, *SE* Spherical equivalence

^*^Comparision among the groups each other LSD-t *P* < 0.05

## Discussion

This study investigated associations of corneal biomechanical properties with the CCT, spherical equivalence, corneal curvature, AL, IOP and age in more details than previous studies. Corneal biomechanical metrics are acknowledged to have a close correlation with CCT [10–14]_._ CH and CRF positively correlated with CCT in our study, thicker CCT was associated with higher CH and CRF. In details, for every 1 μm increasing in CCT was associated with an increase 0.024 mm Hg (*P* < 0.05) in CH and 0.026 mm Hg (*P* < 0.05) in CRF. The standardized regression coefficients suggested that CCT was several times more important than other potential impact factors with CH and CRF. Therefore, we should pay attention to the decreasing in the biomechanical performance of thin corneas. As we know, the thinning of the cornea is the important clinical manifestation of keratoconus. So, is there any difference of corneal biomechanical performance between thin cornea and keratoconus? We refer to the results of Fontes et al. [15] in this study, the corneal biomechanical property of keratoconus was lower compared with the normal thin cornea under the same range of corneal thickness. The result indicated that corneal pathology will affect the corneal biomechanical properties. After we grouped CCT, statistical analysis of the mean values of CH and CRF was conducted to understand the relative normal CH and CRF values corresponding to different corneal thicknesses, in order to provide targeted reference value in clinical work.

In clinical work, the mean value of CH and CRF is usually to evaluate whether the corneal biomechanical properties is normal. If the actual measured value is lower than the mean value, is this meaning that the actual value is abnormal? Our study demonstrated that the CCT is the most important factor to influence the CH and CRF. If the CCT is higher, the CH and CRF will be higher and vice versa. So, we suggest that when we get the CH and CRF value in clinical work, we should firstly refer to the value corresponding CCT sectional range (Table 6). Then, we can just judge whether the corneal biomechanics is normal.

The associations of age with CH, CRF have been argued all the way. An amount of studies showed that age has obviously close relationships with CH, CRF. Narayanaswamy et al. [9] reported that CH and CRF were significantly negative associated with age. Meanwhile, Kirwan et al. [8] reported that there was no statistical difference in children and adults. We found that the lower mean values of CH and CRF in adults group compared with those in children group. It suggested that age had a significant impact on CH and CRF (Table 2). However, an important discovery was found that CCT of children (range, 561.26 ± 38.61um) was significant thicker than adults (range, 538.92 ± 35.98um). Excluding factor of CCT, there were no significant differences about CH and CRF in new children group and new adults group (Table 3). Meanwhile, we discovered that there was no statistical difference among different stages of age with the CH, CRF, CCT (Table 4) from 18 to 48 years old. These findings showed that CH and CRF had no association with age, but CH and CRF were associated with CCT. Narayanaswamy et al. [9] reported that with advancing age, the CH and CRF of Chinese adult decreased; however, we found that study subjects in his research was aged 40 to 83 years while our subjects was aged 8 to 57 years. Obviously, study subjects in his research were older than ours. By further reading literatures, age-related changes of the corneal structure were the direct evidences to explain this phenomenon [16, 17]. Therefore, our study suggests that there was no statistical difference in CH and CRF in young and middle-aged period (age 18–48) with advancing age, but positively associated with the CCT. As stated above, we should not simply think that age is the influence factor of CH and CRF.

The multiple linear regression analysis indicated that both CH and CRF had lower values with the increasing diopter and the AL. Our study demonstrated that the CH and CRF values in high myopic groups were lower than in the low and moderate myopic groups, and there was no statistical difference in the low and moderate myopic groups. The elongation of AL is generally recognized to be connected with changing of sclera structure. Liu et al. [18] considered it resulting from the change of collagen structure, fiber diameter and fiber morphology. As we know, myopic eyes have lower ocular rigidity than normal eyes. As stated by Kotecha [1], corneal biomechanical properties represented the viscoelastic properties of the cornea and mechanical strength of stromal collagen fibrils associating with the extracellular proteoglycan matrix. A remarkable decrease in the diameter of the scleral collagen fibrils and the rate of proteoglycan synthesis were discovered in the increasing of diopter, which will result scleral thinning, the loss of scleral tissue and the decline of scleral mechanical metrics during scleral remodeling [19, 20]. Therefore, cornea may happen similar changes as stated above owing to that corneal stromal layer is the continuation of scleral tissue. As a result, CH and CRF reduce in high myopia. In our study, in different CCT groups, as the thickness of cornea increased, the CH and CRF increased, the difference was statistically significant (*P* < 0.05). In addition, among the three groups with different degrees of myopia, we found that the CH and CRF in the high myopia group were significantly lower than those in the low myopia group, the difference was statistically significant (*P* < 0.05), indicating that with the diopter increased, the CH and CRF decreased. So, this study suggests that whether the CH and CRF are normal in clinical work should not only compare with the mean values of CH and CRF, but also combine with CCT and spherical equivalence together. Moreover, our study firstly proposed the corresponding CCT and spherical equivalence data for clinical reference.

In multiple linear regressions of CH and CRF with corneal curvature, the regression coefficient was 0.174 and 0.156, respectively. Briefly, the low corneal curvature was with lower CH and CRF. These results were consistent with the researches of Hashemi et al.^6^and Hwang et al. [21]. It is well known that the corneal shape influences corneal biomechanical properties. In the structural mechanics, the arch structure was provided with strong compressive resistance, so the steeper of the cornea, the higher of the CH and CRF values. Francis et al. [22] and Matsumoto et al. [23] advocated that the corneal curvature affected corneal rigidity and steeper corneas being more rigid by using dynamic contour tonometry. The more rigid corneas accompanied higher CH and CRF values. However, this finding will cause us to ask why the CH and CRF values reduced in keratoconus patient with high corneal curvature. Through reading articles about keratoconus, especially, Ambekar et al. [24] and Lasagni Vitar et al. [25] reported that the collagen in corneal stroma decreased expression and crosslinked network structure of collagen fibers loosen, which played an important role in the development of keratoconus. Although the thicknesses of individual collagen lamellae are unchanged in keratoconus, but the number of the lamellae in the keratoconus has a significant decline in comparing to normal corneas. Meanwhile, it seems that the regular orthogonal arrangement about individual collagen fibrils within the cone area have been interrupted in the lamellae. These pathological changes lead to progressive thinning, ectasia and successive decline in corneal volume. As stated above, all of these factors combined are likely to explain the reduction of corneal biomechanical properties in keratoconus. What's more, it also shows that the effect of the change in collagen fibrils is more important than corneal curvature.

## Conclusion

In summary, our study reports the association between corneal biomechanical properties measured with the ORA in Chinese myopia and potential impact factors that we mentioned before including CCT, Age, IOP, AL, spherical equivalence and corneal curvature. The CH and CRF represent corneal biomechanical properties. The CH and CRF are shown to be positively associated with CCT, corneal curvature and negatively associated with AL and spherical equivalence. The central corneal thickness was performed to be key factor for affecting the CH and CRF. With advancing age, there was no significant statistical difference in CH and CRF values in young and middle-aged (age 18–48 years) period. In other words, this study proves that the CH and CRF do not change with age in young and middle-aged (age 18–48 years) period, but are mainly close associated with the change of CCT. In view of the importance of corneal biomechanics in the diagnosis of keratoconus, corneal ectasia and other ophthalmopathy in clinical work, our study firstly proposed whether the CH and CRF value of individual patient are normal should refer to the value corresponding CCT sectional range and spherical equivalence. Moreover, our study concluded the corresponding CCT and spherical equivalence data for clinical reference.

## Data Availability

All data generated or analysed during this study are included in this published article.

## Abbreviations

CH: Corneal hysteresis
CRF: Corneal resistance factor
CCT: Central corneal thickness
AL: Axial length
IOP: Intraocular pressure
SE: Spherical equivalence
K: Corneal curvature
ORA: Ocular response analyzer
BCVA: Best corrected visual acuity
D: Diopters
SER: Spherical equivalent refraction
